# The Rational Treatment of Pneumonia

**Published:** 1891-01

**Authors:** 


### THE RATIONAL TREATMENT OF PNEUMONIA.

---

The medical profession hears with languid interest of reports of new and curative drugs in pneumonia. When, however, an ingenious, logical, and sensible application of known physiological data is made to the therapeutics of this disease a more scrutinizing attention is called for. An article by Dr. Andrew H. Smith, on "Acute Obstructive Diseases of the Lungs"(*American Journal of the Medical Sciences*), has all the characters just mentioned. Dr. Smith shows that in an obstructive lung disease like pneumonia, it is the right heart that bears the chief burden. The physicians ought, therefore, to watch it with even more care than the radial pulse. The pulmonary aortic pulse cannot be felt, but its strength and that of the right heart can be gauged by the intensity of the pulmonary aortic valvular sound.

In an obstructive pneumonia the blood is dammed back into the veins, and there is venous congestion, while the arteries are not full enough.

The therapeutist should aim, therefore, to distribute the blood more evenly. This may be done by taking away blood from the veins by venesection. But a safer method is to use such drugs as nitro-glycerine and the other nitrites. Alcohol is also thought to be of great value, not only as a general stimulant and food, but as an aterial depressor. Dr Smith asserts that too much food. espeecially liquid food, should not be given to pneumonia patients, as this imbrrasses digestion and fills up the circulatory system with fluid.

Oxygen gas and artificial respiration are also recommended, to be used even before the patients condition is critical

Digatals should not be used in most cases of pneumonia. This is a dictum supported by good authority and large experience. Yet digatalis contiuues to be given.

Dr. Smith's contribution to the therapeutics of pneumonia is the best that has been made for years. Yet h e makes no allusion to antipyretics or baths. His conclusions are given as follows:

1. In acute pulmoniary obstruction, the danger being from exhaustion of the right heart, the pulse at the wrist does not give reliable indications as to the gravity of the condition.

2. This can be appreciated more correctly by studying the pulmonary circulation by the aid of the pulmonary valve-sound.

3. Marked accentuation of the pulmonary valve-sound indicates a fairly vigorous right heart laboring to overcome resistance in the pulmonary circulation.

4. Decrease of a previously existing accentuation, with only moderate dyspnœa, indicates decrease of pulmonar obstruction.

5. Decrease of accentation, with increase of respiratory distress, indicates that the right heart is becoming exhausted.

6. Relief is to be sought: *a*, by regulating the diet in conformity with the diminished power of digestion and sanguinification; *b*, by the use of medicines which dilate the arteries and promote transferrence of blood to them from the vains; *c*, by the inhalation of oxygen gas, *d*, by artificial respiration; *e*, by placing ligatures about the extremeties in order to retain the blood in them and prevent its return to the heart.—*Medical Record.*

